# Supplementary material for: NN-RNALoc: Neural network-based model for prediction of mRNA sub-cellular localization using distance-based sub-sequence profiles
Source: PLoS One. 2023 Sep 14;18(9):e0258793. doi: 10.1371/journal.pone.0258793 (PMC10501558; doi:10.1371/journal.pone.0258793)
Supplement: S1 Table — (PDF) [file pone.0258793.s001.pdf]

S1 Table: Results of 30 times 10-fold cross-validation of NN-RNALoc (with and without employing PPI information) compared with RNATracker(fixed length mode) and mRNALoc on the human part of RNALocate dataset.

Methods	NN-RNALoc					NN-RNALoc(noPPI)					RNATracker					mRNALoc				
Criteria	Cyt	ER	EX	Mit	Nuc	Cyt	ER	EX	Mit	Nuc	Cyt	ER	EX	Mit	Nuc	Cyt	ER	EX	Mit	Nuc
Precision	**0.74**	**0.56**	**0.91**	0.00	0.52	0.65	0.40	0.00	0.00	**0.61**	0.66	0.52	0.00	0.00	0.49	0.74	0.46	0.00	0.00	0.52
Recall	0.72	**0.48**	**0.04**	0.00	0.70	**0.84**	0.14	0.00	0.00	0.51	0.59	0.06	0.00	0.00	0.54	0.61	0.50	0.00	0.00	**0.72**
F-score	**0.74**	**0.52**	**0.07**	0.00	0.60	0.73	0.21	0.00	0.00	0.56	0.73	0.11	0.00	0.00	0.53	0.67	0.48	0.00	0.00	**0.61**
Average Accuracy	**0.65**					0.63					0.63					0.61				
Average MCC	**0.40**					0.30					0.34					0.37				

The names of compartments are abbreviated as Cyt : Cytosol, ER: Endoplasmic Reticulum, EX : Extracellular Region, Mit :Mitochondria, Nuc: Nucleus.
