# Supplementary material for: NN-RNALoc: Neural network-based model for prediction of mRNA sub-cellular localization using distance-based sub-sequence profiles
Source: PLoS One. 2023 Sep 14;18(9):e0258793. doi: 10.1371/journal.pone.0258793 (PMC10501558; doi:10.1371/journal.pone.0258793)
Supplement: S2 Table — (PDF) [file pone.0258793.s002.pdf]

S2 Table: Results of AUC-ROC (ROC) and AUC-PR (PR) for each location of human part of RNALocate database obtained by NN-RNALoc, SVM-RNALoc, RF-RNALoc, XGBoost-RNALoc, DNN-RNALoc, LightGBM-RNALoc.

Methods	NN-RNALoc		SVM-RNALoc		RF-RNALoc		XGBoost-RNALoc		LightGBM-RNALoc	
Criteria	ROC	PR	ROC	PR	ROC	PR	ROC	PR	ROC	PR
Cyt	**0.76**	**0.66**	0.65	0.58	0.65	0.55	0.66	0.28	0.45	0.28
ER	**0.70**	**0.79**	0.23	0.31	0.55	0.40	0.34	0.42	0.44	0.38
EX	**0.65**	**0.63**	0.00	0.00	0.00	0.00	0.00	0.00	0.00	0.00
Mit	0.00	0.00	0.00	0.00	0.00	0.00	0.00	0.00	0.00	0.00
Nuc	**0.71**	**0.70**	0.34	0.50	0.25	0.40	0.33	0.45	0.48	0.38

The names of compartments are abbreviated as Cyt : Cytosol, ER: Endoplasmic Reticulum, EX : Extracellular Region, Mit :Mitochondria, Nuc: Nucleus. NN-RNALoc (with employing NN on k-mer and distance-based profiles features); SVM-RNALoc (with employing support vector machine on k-mer and distance-based profiles features); XGBoost-RNALoc(with employing extreme gradient boosting on k-mer and distance-based profiles features); LightGBM-RNALoc (with employing light gradient-boosting machine on k-mer and distance-based profiles features).
