# Supplementary material for: NN-RNALoc: Neural network-based model for prediction of mRNA sub-cellular localization using distance-based sub-sequence profiles
Source: PLoS One. 2023 Sep 14;18(9):e0258793. doi: 10.1371/journal.pone.0258793 (PMC10501558; doi:10.1371/journal.pone.0258793)
Supplement: Attachment — Submitted filename: Response to Reviewers.pdf [file pone.0258793.s003.pdf]

Response to the reviewers

Manuscript title:

NN-RNALoc: neural network-based model for prediction of mRNA sub-cellular localization using distance-based sub-sequence profiles

Manuscript number: PONE-D-21-31782

Revision Version: 1

We thank the reviewers for their critical assessment of our work. In the following we address their concerns point by point.

Reviewer 1

Reviewer Point 1.1 — *The text has many grammatical and spelling errors, which need to be addressed to be able to better appreciate the work presented. For instance, "dimension" and "distance" are misspelled on line 57 and the Figure 2 legend, respectively.*

Reply: We thank the reviewer for this comment. The authors carefully read the entire article, corrected the typos, and rewrote numerous sections and paragraphs to improve the manuscript's readability. In addition, a native English speaker assisted us in revising the manuscript. In blue, the responses to the reviewer's comments are added to the manuscript.

Reviewer Point 1.2 — *The authors spend a considerably large amount of text on the introduction which spans, 144 lines of text, over 2 pages even enumerating machine learning guidelines from a previous study. I would recommend the introduction be condensed into more concise text which would make the transition to methods and results smoother.*

Reply: We thank the reviewer for pointing us to this structural point. The section has been revised to be more concise.

Reviewer Point 1.3 — *Why is a distance-based sub-sequence of k=2 optimal, why not larger values? It seems like k=2 is capturing information already present in the k-mers counts and would be interesting to hear the authors discuss their methodology for selecting k=2.*

Reply: We appreciate your noticing this. We believe that our explanation was unclear. To eliminate the confusion, we add Figure 2 and also revised Fig 3 in the old manuscript to

more accurately depict our distance-based profile. We added the following paragraph and also edited Figure 3 to be more clear in the "Feature encoding" section of the manuscript;

The main drawback of k-mer representation is that when k increases, the feature vector becomes extremely large and sparse, which can be memory-inefficient and can reduce the performance of the model. In order to mitigate the issue of small repeat regions, it may be advantageous to employ larger k-mer sizes. However, as the number of matching subsequences decreases, large k-mers become computationally infeasible and result in a significant sparsity in the feature vector. In this study, we propose a novel distance-based representation to partially address this issue. In the novel distance-based profiles, the distance between the first and last nucleotide of the subsequence that we counted is k. The frequency of this subsequence is then determined for each pair of nucleotides separated by k. Consequently, for an mRNA sequence S and a distance k, the following 16-element feature vector is obtained: $D_k(S) = [w_1, w_2, ..., w_{16}]$, where w_i is the frequency of each distance-based sub-sequence and X is a sub-sequence of size k. For any k, an illustration of all subsequences to count is provided in Figure 2.

w_1: AXA	w_2: AXC	w_3: AXG	w_4: AXT
w_5: CXA	w_6: CXC	w_7: CXG	w_8: CXT
w_9: GXA	w_{10}: GXC	w_{11}: GXG	w_{12}: GXT
w_{13}:TXA	w_{14}: TXC	w_{15}: TXG	w_{16}: TXT

Fig. 2: For an mRNA sequence S and a distance k, we depict the 16-element feature vector, where w_i is the frequency of each distance-based subsequence and X denotes a possible sub-sequences of size k.

It is obvious that for an mRNA sequence S with a length of m, X can be replaced with a sub-sequence of nucleotides (A, G, C, and T) ranging from size 0 to m-2. As an example, let's consider S to be the mRNA with the sequence ACGCCGC with a length of 7, so X can be a sub-sequence of maximum size 5. For example, in Figure 3, four distance-based substructures of ACGCCGC are shown in three different colors. The two sub-sequences CGCC and CCGC with distance 2 are shown in green, one sub-sequence GCCGC with distance 3 is drawn in red, and one sub-sequence ACGCCGC with distance 5 is illustrated in black. Also, if we want, for instance, to calculate w_3 and w_6 in this sequence, for w_3 : AXG, we have one sub-sequence ACG (k=1) and one sub-sequence ACGCCG (k=4), so the frequency of w_3 is 2. For w_6 : CXC, the sequence contains one sub-sequence CC (k=0), two sub-sequences CGC (k=1), one sub-sequence CCGC (k=2), one sub-sequence CGCC (k=2), and one sub-sequence CGCCGC (k=4), therefore the frequency of w_6 is 6. In this work, we tested a wide range of distances, and after many trials and errors, we found the best range for k to be between 0 and 8. As a result, the length of the created feature vector is $9 \times 16 = 144$.

Reviewer Point 1.4 — *In Table 3 and 4, two benchmarks are performed, however, the authors utilize two different metrics for evaluation. Table 3 is correlation based while*

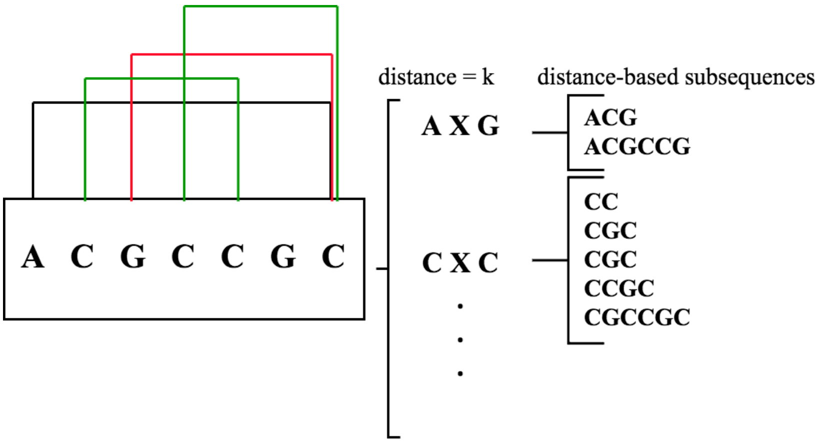

Fig. 3: Illustrating distance-based profiles. Four distance-based substructures are shown in three different colors for the mRNA sequence S=ACGCCGC. Two sub-sequences CGCC and CCGC with k=2 are shown in green, one sub-sequence GCCGC with k=3 is depicted in red, and one sub-sequence ACGCCGC with k=5 is illustrated in black. In addition, the figure depicts the possible subsequences of S between A and G (AXG) and C and C (CXC).

Table 4 uses the standard multi-class accuracy metrics. This is slightly confusing because they are all performing the same classification tasks, the metrics used should be the same between benchmarks to enable better comparisons.

Reply: We thank the reviewer for their notice. Our methodology has been evaluated using distinct datasets, as stated in the paper. The first method, known as Cefra-seq, uses a real number to represent the amount of gene expression in each of the four compartments. Therefore, Pearson and Spearman correlations were employed to assess the performance of the models in this data set. The second dataset, compiled from the RNALocate database, is among the most commonly used datasets for RNA localization. The element information of this data set is a binary vector indicating whether a specific RNA is present at a given location. Given that this data set considers five locations, the length of this binary vector is also five. We use a classification method within this data set to predict the localization of an RNA. This data set necessitates the classification metrics, precision, recall, f-score, MCC, and ACC for comparing different classification methods. For better comprehension, we added the following paragraph to the "Evaluation criteria" subsection and Table 3 in the "Results" section of the manuscript;

As stated previously, we work with two datasets, and due to the differences in their structures, we compare different metrics to evaluate the performance of the model on each dataset. As described previously, the CeFra-Seq localization values are continuous. We therefore consider correlation measurements when evaluating model performance similar to [9] study. The initial measure is Pearson Correlation. Pearson Correlation is a method for measuring the linear correlation between predicted and observed values. It has a value

between 1 and -1, with +1 representing a total positive linear correlation, 0 representing no linear correlation, and -1 representing a total negative linear correlation. In order to better evaluate the performance of the model, we also consider the Spearman correlation between predicted and experimental values to capture the order of locations to which an mRNA belongs. In addition, we employ classification metrics in the RNALocate dataset because localization information is discrete values similar to 12 study.

In Table 3, we have summarized all the metrics used to evaluate the performance of the models on the two benchmarks.

Table 3: **A summary of the localization information for two datasets and metrics used to assess models performance.**

Dataset	Localization information	Metrics
CefraSeq	Normalized gene expression valuess	Regression metrics: Pearson Cor. and Spearman Cor
RNALoc	Single location	Classification metrics: Precision, ACC, F-score, MCC

We use correlation measurements for CefraSeq, and classification metrics for RNALocate dataset. Pearson Cor: Pearson Correlation; Spearman Cor: Spearman Correlation; ACC: Accuracy; MCC: Matthews Correlation Coefficient.

Reviewer Point 1.5 — *The authors state the advantages of their distance-based sub-sequence profiles many times but do not directly quantify their benefits. It would be informative for the authors to create a new model only using k-mers then they can compare the accuracies of this model to the NN-RNALoc(noPPI) model to directly estimate the effects of their new distance-based sub-sequence profiles. This would allow the visualization of the increases in accuracy from k-mers, PPI and distance-based features.*

Reply: We thank the reviewer very much for considering this claiming point. In Table 4, we added the experiment using only k-mer frequencies and compared the results to the case when the distance-based profiles are also included. We have updated the Table 4 and added the following paragraphs in the "Result" section of the manuscript;

DNN-kMer is a multilayer perceptron-based predictor that extracts k-mer features from sequences (1-mers to k-mers). In both data sets, the DNN-kMer model was trained on 1-mers to 8-mers, and the best results were obtained when all 1-mer to 5-mer information was taken into account. Therefore, DNN-5mer's inputs are a 1364-dimensional ($4^1+4^2+4^3+4^4+4^5$) vector. As a result, using 1-mers to 5-mers as features, we evaluate the performance of NN-RNALoc and DNN-5mer. DNN-5mer has only two hidden layers with the same number of neurons as the input vector. In the hidden layer, the Relu activation function is utilized. Despite the fact that both NN-RNALoc and DNN-5mer have a simple architecture, DNN-5mer performs significantly worse, with Pearson correlations of 0.63 in the Membrane, 0.55 in Insoluble, 0.42 in the Membrane, and 0.48 in the nucleus. Overall, NN-RNALoc achieves a Pearson correlation approximately 35% higher than DNN-5mer. In addition, we ran NN-RNALoc with only k-mer frequencies (for k from 1 to 5) to evaluate the effect of incorporating

the distance-based profile into the model. As Table 4 represents, in this context (comparing NN-RNALoc(no PPI) and NN-RNALoc(k-mer profile)) the Pearson correlations were 9% lower in total, demonstrating the advantages of using distance-based profiles.

Table 4: **Average Pearson correlations of 30 times 10-fold cross-validation in each location of Cefra-Seq dataset obtained by different methods.**

Location	NN-RNALoc	NN-RNALoc(no PPI)	NN-RNALoc(k-mer profile)	RNATracker fixed	RNATracker full	DNN-5mer
Cytosol	0.69	0.67	0.66	0.68	**0.70**	0.63
Insoluble	**0.65**	0.61	0.60	0.62	0.64	0.55
Membrane	**0.54**	0.52	0.47	0.47	**0.54**	0.42
Nuclear	**0.55**	0.52	0.50	0.49	0.54	0.48

NN-RNALoc (with employing PPI, k-mer and distance-based profiles);
NN-RNALoc(no PPI) (k-mer and distance-based profiles); NN-RNALoc(only k-mer);
RNATracker (fixed length mode); RNATracker full (full length mode); DNN-5mer
(1-mers to 5-mers)

Reviewer Point 1.6 — *The utilization of novel features to improve classifier accuracy is very interesting, however it would be equally intriguing to see why these features increase accuracy. For example, what are the most informative distance-based subsequence profiles for each subcellular location? Are some of these, or their respective k-mers enriched for RNA-binding motifs? In addition, are certain subcellular locations enriched for certain protein-protein interactions? I would recommend adding a figure exploring these questions.*

Reply: First of all, thank you for considering this point, which paves the way for future results in this field. We did the experiment in order to assess the importance of each k-mer profile and added the following paragraphs and also Fig 5, Fig 6 and Fig 7 to the manuscript for better clarification;

To evaluate the effect of incorporating PPI information into our model, the following analysis was performed on both the CeFra-Seq and RNALocate datasets. We only utilize 5-mer and also distance-based sub-sequence information derived from mRNA sequences and compare the results to the scenario in which PPI information is also incorporated into the model. When the reduced PPI matrix is used in the model for the Cefra-se dataset, NN-RNALoc achieves almost 11% higher Pearson correlation in total for all locations, as shown in Table 4. We conduct the same analysis on the RNALocate dataset and human-related transcripts too, utilizing only sequence-based information in the model. These results, which are the same as those found in the first dataset, also show that when NN-RNALoc uses PPI information in the second dataset, its performance totally improves with 10% increase in MCC and 2% in accuracy.

Fig 5 and Fig 6 compile the results for a more precise comparison of the performance of the NN-RNALoc algorithm with PPI information (NN-RNALoc) and without PPI information (NN-RNALoc(no PPI)) besides other methods. Fig 5 displays the resulted average of Pearson

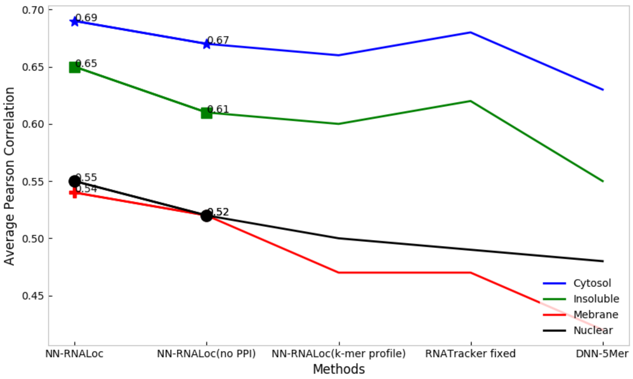

Fig. 5: Comparison of Pearson correlations values of NN-RNALoc algorithm with other methods for Cefra-Seq dataset.

correlation for the CeFra-Seq dataset for four locations, and Fig 6 shows the average of F-score values for the five locations in the RNALocate dataset. According to Fig 5 and Fig 6, considering PPI information improves the results for all locations in both datasets and has the greatest influence on predicting the insoluble location in CeFra-Seq dataset and Endoplasmic Reticulum location in the RNALocate dataset. To evaluate the impact of including distance-based profiles in the model, we omit this information from the feature vector. As previously discussed in the results and as shown in Table 4, Fig 5 and Fig 6, the poorer performance of NN-RNALoc on both datasets when only k-mer frequencies (for k from 1 to 5) are used can potentially demonstrate the impact of distance-based profiles. Finally, for a more detailed evaluation and to determine the impact of each distance-based k-mer on the prediction of mRNA location, the following experiment was conducted on the CeFra-Seq dataset. We independently considered each distance-based profile for k ranging from 0 to 8. Fig 7 depicts the average Pearson correlation in each of four locations when a single distance-based k-mer profile was used. Using 8-mer distance-based profiles yields the highest correlation in Cytosol, Insoluble, and Nuclear, which are represented by blue, orange, and green curves, respectively, as shown in Fig 7. However, for Membrane, which is depicted by a red curve, the highest correlation is obtained using a 4-mer distance-based profile, despite the fact that the differences in Pearson correlations are negligible. Therefore, in order to find all possible patterns in mRNA sequences, we decided to look at the combination of distance-based profiles for all k-mers in the range of 0 to 8.

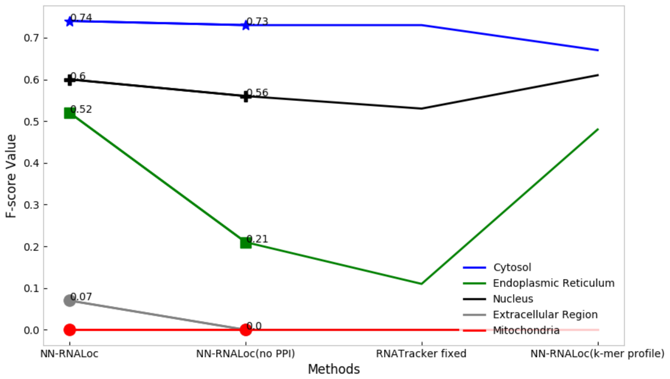

Fig. 6: The average of F-score values for the five locations in the RNALocate dataset.

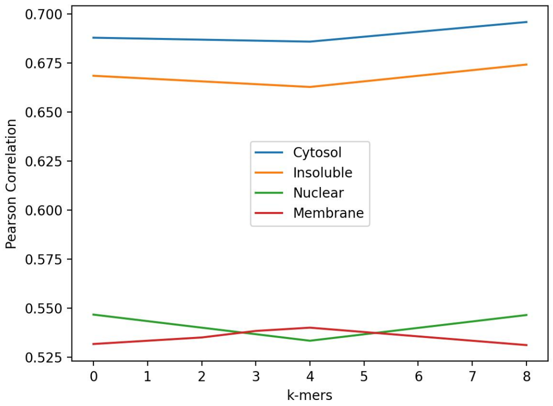

Fig. 7: Impact of each distance-based k-mer in each location. Pearson correlation obtained by NN-RNALoc on CeFra-Seq dataset when employing each distance-based profile for k in range 0 and 8, individually. Four locations are represented in four different colors; blue: Cytosol, orange: Insoluble, green: Nuclear, red: Membrane.

Reviewer 2

Reviewer Point 2.1 — *Authors proposed a deep learning framework for mRNA sub-cellular locations prediction. Authors have proved that information of proteins assists model to predict sub-cellular locations more precisely. The paper seems interesting and will be helpful for biomedical researchers.*

Reply: We thank the reviewer for his/her time and kind review.
