# Supplementary material for: NN-RNALoc: Neural network-based model for prediction of mRNA sub-cellular localization using distance-based sub-sequence profiles
Source: PLoS One. 2023 Sep 14;18(9):e0258793. doi: 10.1371/journal.pone.0258793 (PMC10501558; doi:10.1371/journal.pone.0258793)
Supplement: Attachment — Submitted filename: response.pdf [file pone.0258793.s004.pdf]

# Response to the reviewers

**21Dec2022**

**Manuscript title:**

NN-RNALoc: neural network-based model for prediction of mRNA sub-cellular localization using distance-based sub-sequence profiles

**Manuscript number:** PONE-D-21-31782

**Revision Version**: 2

We thank the reviewer for the critical assessment of our work. In the following, we address the concerns point by point.

---

# Reviewer 1

**Reviewer Point 1.1** — *In this study, the authors developed an ANN based computational model for localization prediction of mRNA. Following are my major concerns that need to be addressed before acceptance.*

**Reply**: We would like to express our gratitude for the reviewer's insightful and helpful comments. We have responded to all comments and suggestions and conducted several new analyses, and we believe the manuscript's quality has been significantly enhanced. The responses to the comments are listed below.

**Reviewer Point 1.2** — *The authors should cite the existing work on mRNA localization. Following articles must be cited.*
*[r1] Asim, M.N., Ibrahim, M.A., Malik, M.I., Zehe, C., Cloarec, O., Trygg, J., Dengel, A. and Ahmed, S., 2022. EL-RMLocNet: An explainable LSTM network for RNA-associated multi-compartment localization prediction. Computational and Structural Biotechnology Journal.*
*[r2]Meher, P.K., Rai, A. and Rao, A.R., 2021. mLoc-mRNA: predicting multiple sub-cellular localization of mRNAs using random forest algorithm coupled with feature selection via elastic net. BMC bioinformatics, 22(1), pp.1-24.*
*[r3] Wang, D., Zhang, Z., Jiang, Y., Mao, Z., Wang, D., Lin, H. and Xu, D., 2021. DM3Loc: multi-label mRNA subcellular localization prediction and analysis based on multi-head self-attention mechanism. Nucleic Acids Research, 49(8), pp.e46- e46.*
*[r4] Zhang, Z.Y., Yang, Y.H., Ding, H., Wang, D., Chen, W. and Lin, H., 2021. Design powerful predictor for mRNA subcellular location prediction in Homo sapiens. Briefings in Bioinformatics, 22(1), pp.526-535.*

*[r5] Yan, Z., Lecuyer, E. and Blanchette, M., 2019. Prediction of mRNA subcellular localization using deep recurrent neural networks. Bioinformatics, 35(14), pp.i333-i342.*

**Reply**: We thank the reviewer for this comment. References r4 and r5 were cited in the previous version of the manuscript in the introduction section as follows:
In recent years, computational predictors have emerged that rely heavily on machine learning techniques [r4].
Zhang et al., developed a computational method, iLoc-mRNA, which was trained on the RNALocate dataset and applied an SVM model for multiclass classification [r4].
On the other hand, mRNA localization has been studied for many years. There are two well-known experimental datasets in this regard: cell fractionation with RNA-sequencing (CeFra-Seq) and APEX-RIP [r5].
RNATracker [r5] was the first mRNA localization prediction model to be developed in 2019. RNATracker predicts the location of mRNAs in CeFra-Seq and APEX-RIP datasets using convolutional neural network (CNN) and long short-term Memory (LSTM).

The references r1, r2, and r3 now are added in the revised paper (references 15, 16, and 17 in the paper) in the "Introduction" section in the following paragraph:

"Meher et al. presented "mLoc-mRNA" to forecast nine distinct sub-cellular localizations for mRNAs. They used k-mers of sizes 1-6 to transform each mRNA sequence into a numerical feature vector. They applied the Elastic Net statistical model to extract the best features from the k-mer features. The sub-cellular localization of mRNAs was then predicted using a Random Forest classifier [r2]. In 2021, a multi-label mRNA sub-cellular localization predictor named "DM3Loc" was also proposed using Deep Learning, which predicts the 6 distinct locations of mRNAs in Homo sapiens. They prepared data as the input for CNN using mRNA sequences as the raw data and a novel multi-head self-attention mechanism capable of producing sequence motifs [r3]. The deep learning model "EL-RMLocNet", which predicts the subcellular localization of four different RNA classes (mRNA, miRNA, lncRNA, and snoRNA) in Homo sapiens and Mus musculus species, was developed in [r1]. To identify the most informative features from raw RNA sequences, they used the LSTM network, which captured the short and long range relations of nucleotide k-mers."

**Reviewer Point 1.3** — *The authors compared the accuracy with only two existing tools such as RNATracker and mRNALoc. The other tools (mentioned in comment 1) should also be considered to claim the superiority of the NN-RNALoc.*

**Reply**: We originally submitted this manuscript in 10/2021 and it has been under review since then. Back then, RNATracker and mRNALoc were the two main methods that were available for us to benchmark. As a result, we have not included DM3Loc, iLoc-mRNA, mLoc-mRNA, and EL-RMLocNet in our paper. We appreciate the reviewer's attention to this point. In response to this feedback, we used the RNALocate dataset and benchmarked our algorithm using this dataset. The RNALocate dataset is the most well-known dataset in this field and was used for validation for all the algorithms mentioned in the previous studies. Several performance metrics were computed, and our approach was compared to those described in comment 1.

In the revised manuscript, new tables (Tables 5 and 6) and the following paragraphs are added to the new version of the manuscript:

The following paragraph is added in the "Abstract" section:

"On two benchmark datasets, CeFra-Seq and RNALocate, the performance of NN-RNALoc is compared to powerful predictive models proposed in previous works (mRNALoc, RNA-Tracker, mLoc-mRNA, DM3Loc, iLoc-mRNA, and EL-RMLocNet), and a ground neural (DNN5-mer) network. Compared to the previous methods, NN-RNALoc significantly reduces computation time and also outperforms them in terms of accuracy."

The following sentences are added in the "Introduction" section:

"In this study, we focus on the CeFra-Seq and RNALocate datasets, as well as powerful predictive models including mRNALoc, RNATracker, mLoc-mRNA, DM3Loc, iLoc-mRNA, and EL-RMLocNet as benchmarks."

"In the Results section, we describe the performance of NN-RNALoc on the aforementioned two datasets and compare it to different methods: mRNALoc, RNATracker, DNN-5mer, DM3Loc, iLoc-mRNA, mLoc-mRNA, and EL-RMLocNet.

The following paragraph is added in the "Materials and methods" section:

The RNALocate sub-cellular localization data were obtained from RNALocate at `https://www.rna-society.org/rnalocate/`. The sequences of mRNAs were downloaded from GenBank and the mRNA sequence data in the FASTA format were obtained from the NCBI on December 2022 [24]. In total, this dataset contains 11,180 mRNAs, of which 5,905 are human transcripts and 5,275 are non-human transcripts. Table 1 provides a summary of this dataset. Notably, because the data produced by APEX-RIP is fairly noisy [9,12], we did not use it in this study."

**Table 1: Total number of mRNAs in each five locations in the RNALocate dataset.**

| Location | Human Species | Non-human Species |
|---|---|---|
| Cytoplasm | 3,427 | 1,534 |
| Endoplasmic Reticulum | 1,173 | 8 |
| Extracellular Region | 26 | 509 |
| Mitochondria | 5 | 344 |
| Nuclear | 1,274 | 2,880 |
| Total | 5,905 | 5,275 |

The first column represents each cellular compartment. The second and third columns reveal the number of human and non-human transcripts, respectively.

The following paragraph is added in the "Result" section:

"RNALocate is the most well-known dataset in this field and was used for validation for all the algorithms mentioned in the previous studies. The performance of NN-RNALoc on RNALocate is benchmarked against RNATracker, DM3Loc, mRNALoc, iLoc-mRNA, EL-RMLocNet, and mLoc-mRNA methods. We report the area under the Receiver Operator Characteristic (ROC) curve (AUC-ROC) and the area under the Precision-Recall (PR) curve

(AUC-PR) for a fair comparison of the tested methods similar to RNATracker, DM3Loc, mRNALoc, iLoc-mRNA, EL-RMLocNet, and mLoc-mRNA studies. Table 5 summarizes the AUC-ROC, AUC-PR, and Average MCC for different methods for the human part of the RNALocate dataset. For Cyt location, NN-RNALoc and mRNALoc outperformed others based on AUC-ROC and AUC-PR, respectively. For ER, iLoc-mRNA and NN-RNALoc outperformed others based on AUC-ROC and AUC-PR, respectively. For EX, mLoc-mRNA and RNATracker outperformed others based on AUC-ROC and AUC-PR, respectively. For the Nuc location, mLoc-mRNA and RNATracker outperformed others based on AUC-ROC and AUC-PR, respectively. As seen in Table 5, none of the methods outperform the other methods in all locations and for Cyt and ER locations, NN-RNALoc outperformed well-known methods. Similar to some previous methods, we only considered single-location mRNA sequences in the RNALocate dataset. Except for DM3Lo and mLocmRNA methods, which predict multiple locations for each mRNA sequence, all other methods only predict a single location. If the actual location of an mRNA sequence was presented in the prediction results of the mLocmRNA and DM3Lo methods, it was reported as a true prediction. It is obvious that by predicting multiple locations, these methods improve the performance of their algorithm in some locations compared to other methods, as shown in Table 5. Similarly, Table 6 represents the result of different methods on the non-human part of the RNALocate dataset. In this case, NN-RNALoc outperformed existing methods for the Nuc location and obtained nearly similar results to other methods. In terms of average MCC, NN-RNALoc performs better than other methods, which shows that our method works well overall.

**Reviewer Point 1.4** — *Here are several shallow learning (SVM, Random forest, XGBoost, LightGBM, etc.) and deep learning models are available. The performance of ANN (used in this study) should be compared with these methods as well.*

**Reply**: We appreciate the reviewer's suggestion and concur with the reviewer that shallow learning models should be used to benchmark our approach. On the other hand, we would like to point out that shallow algorithms are used within the benchmarked algorithms. For example, mLoc-mRNA uses a Random Forest classifier to predict the subcellular localization of mRNA. In mRNALoc, SVM is used as the learning algorithm. EL-RMLocNet and DM3Loc use Deep Learning to predict the subcellular localization of mRNAs. In accordance with comment 2, we have benchmarked our approach with the mentioned algorithms.

Also, based on reviewers' recommendations, we have reported the results of our algorithm utilizing several shallow learning methods (SVM, Random forest, XGBoost, and LightGBM). The following paragraph and Tables 7 and S2 are added in the "Result" section to describe this part of the work:

" In addition, we used other shallow learning algorithms e.g. SVM, RF, Extreme Gradient Boosting (XGBoost), and light gradient-boosting machine (LGBM) [39] for our learning process methods instead of using NN. SVM-RNALoc used SVM on k-mer and distance-based profile features, XGBoost-RNALoc employed XGBoost on k-mer and distance-based profile features, and LightGBM-RNALoc applied LightGBM on k-mer and distance-based profile features. Table 7 and S2 indicate the results of these algorithms for the Cefra-Seq and RNALocate datasets, respectively. The results show that NN-RNALoc for most locations

**Table 5: Results of AUC-ROC and AUC-PR for different methods on the human part of the RNALocate dataset.**

| Method | Compartment | AUC-ROC | AUC-PR | Average MCC |
|---|---|---|---|---|
| NN-RNALoc | Cyt | **0.76** | 0.71 | |
| | ER | 0.71 | **0.79** | |
| | EX | 0.65 | 0.63 | **0.40** |
| | Mit | 0 | 0 | |
| | Nuc | 0.79 | 0.77 | |
| NN-RNALoc (noPPI) | Cyt | 0.73 | 0.67 | |
| | ER | 0.66 | 0.55 | |
| | EX | 0 | 0 | 0.30 |
| | Mit | 0 | 0 | |
| | Nuc | 0.70 | 0.74 | |
| RNATracker | Cyt | 0.73 | 0.31 | |
| | ER | 0.62 | 0.18 | |
| | EX | 0.75 | **0.99** | 0.34 |
| | Mit | 0 | 0 | |
| | Nuc | 0.75 | **0.86** | |
| DM3Loc | Cyt | 0.74 | 0.31 | |
| | ER | 0.69 | 0.25 | |
| | EX | 0 | 0 | 0.24 |
| | Mit | 0 | 0 | |
| | Nuc | 0.77 | 0.87 | |
| mRNALoc | Cyt | 0.60 | **0.76** | |
| | ER | 0.37 | 0.14 | |
| | EX | 0.40 | 0.98 | 0.37 |
| | Mit | 0 | 0 | |
| | Nuc | 0.60 | 0.76 | |
| iLoc-mRNA | Cyt | 0.51 | 0.72 | |
| | ER | **0.81** | 0.57 | |
| | EX | 0 | 0 | 0.20 |
| | Mit | 0 | 0 | |
| | Nuc | 0.51 | 0.72 | |
| EL-RMLocNet | Cyt | 0.74 | 0.45 | |
| | ER | 0 | 0 | |
| | EX | 0.75 | 0.67 | 0.38 |
| | Mit | 0 | 0 | |
| | Nuc | 0.68 | 0.56 | |
| mLoc-mRNA | Cyt | 0.75 | 0.71 | |
| | ER | 0.75 | 0.72 | |
| | EX | **0.76** | 0.77 | 0.38 |
| | Mit | 0.98 | 0.99 | |
| | Nuc | **0.80** | 0.79 | |

outperforms other methods. Hence, we used the NN method to predict locations based on k-mer and distance-based profile features. Moreover, we applied the DNN-kMer method which is a multilayer perceptron-based predictor that extracts k-mer features from sequences (1-mers to k-mers) and compared them with NN-RNALoc (please see Table 4). The results show that NN-RNALoc outperforms the other shallow learning approaches."

**Reviewer Point 1.5** — *The NN-RNALoc can predict an mRNA to any one localization. However, it is the very fact that a single mRNA could be present in more than one location. So, how the proposed study will address this problem?*

**Table 6: Results of AUC-ROC and AUC-PR for different methods on the non-human part of the RNALocate dataset.**

| Method | NN-RNALoc | | | | | RNATracker | | | | | mRNALoc | | | | | iLoc-mRNA | | | | | EL-RMLocNet | | | | |
|---|---|---|---|---|---|---|---|---|---|---|---|---|---|---|---|---|---|---|---|---|---|---|---|---|---|
| Compartment | Cyt | ER | EX | Mit | Nuc | Cyt | ER | EX | Mit | Nuc | Cyt | ER | EX | Mit | Nuc | Cyt | ER | EX | Mit | Nuc | Cyt | ER | EX | Mit | Nuc |
| AUC-ROC | 0.71 | 0 | 0.4 | 0.71 | 0.54 | **0.77** | 0 | 0.45 | **0.9** | 0.68 | 0.71 | 0.63 | **0.48** | 0.76 | 0.44 | 0.23 | **0.65** | 0 | 0 | **0.69** | 0.73 | 0 | 0 | 0.7 | 0.78 |
| AUC-PR | 0.77 | 0 | 0.38 | 0.93 | **0.72** | 0.69 | 0 | **0.5** | 0.85 | 0.7 | 0.57 | 0.1 | 0.23 | **0.99** | 0.71 | 0.16 | 0.48 | 0 | 0 | 0.56 | **0.8** | 0 | 0 | 0.59 | 0.68 |
| MCC | **0.55** | | | | | 0.43 | | | | | 0.47 | | | | | 0.38 | | | | | 0.5 | | | | |

The names of compartments are abbreviated as Cyt : Cytosol, ER: Endoplasmic Reticulum, EX : Extracellular Region, Mit :Mitochondria, Nuc: Nucleus.

**Table 7: Average Pearson correlations of 30 times 10-fold cross-validation in each location of Cefra-Seq dataset obtained by NN-RNALoc, SVM-RNALoc, RF-RNALoc, XGBoost-RNALoc, DNN-RNALoc, LGBM-RNALoc.**

| Location | NN-RNALoc | SVM-RNALoc | RF-RNALoc | XGBoost-RNALoc | LGBM-RNALoc |
|---|---|---|---|---|---|
| Cytosol | 0.69 | 0.65 | **0.77** | 0.45 | 0.65 |
| Insoluble | **0.65** | 0.43 | 0.37 | 0.56 | 0.33 |
| Membrane | **0.54** | 0.33 | 0.45 | 0.36 | 0.45 |
| Nuclear | **0.52** | 0.35 | 0.43 | 0.47 | 0.42 |

NN-RNALoc (with employing NN on k-mer and distance-based profiles features); SVM-RNALoc (with employing support vector machine on k-mer and distance-based profiles features); XGBoost-RNALoc(with employing extreme gradient boosting on k-mer and distance-based profiles features); LGBM-RNALoc (with employing light gradient-boosting machine on k-mer and distance-based profiles features).

**Reply**: As the reviewer correctly pointed out, a single mRNA could be present in more than one location and our method can predict more than one location for a single mRNA. The following paragraph is added in the "Discussion" section to address this comment:

" Our method has been evaluated using two different datasets. The first dataset, CeFra-Seq, uses a continuous set of values to represent the localization probability of each of the four compartments. Hence, we predict a probability value for each compartment of this dataset. Then, we use Pearson and Spearman correlations to assess the performance of the models in the CeFra-Seq dataset. Using our method, we can either select one location using the maximum probability value or select multiple locations by setting a probability threshold. The second dataset, compiled from the RNALocate dataset, is among the most commonly used datasets for RNA localization and all methods applied for the comparison report their results on this dataset. The element information of this dataset is a binary vector indicating whether a specific RNA is present at a given location or not. Given that this dataset contains five locations, the length of this binary vector is also five. We use a classification method on this dataset to predict the localization of a given mRNA. For evaluating the performance of the classification algorithms, precision, recall, f-score, MCC, and ACC were used. We also reported AUC-ROC and AUC-PR for classification performance comparisons. It is crucial

**Table S2: Results of AUC-ROC (ROC) and AUC-PR (PR) for each location of the human part of RNALocate dataset obtained by NN-RNALoc, SVM-RNALoc, RF-RNALoc, XGBoost-RNALoc, DNN-RNALoc, LightGBM-RNALoc.**

| Methods | NN-RNALoc | | SVM-RNALoc | | RF-RNALoc | | XGBoost-RNALoc | | LightGBM-RNALoc | |
|---|---|---|---|---|---|---|---|---|---|---|
| Criteria | ROC | PR | ROC | PR | ROC | PR | ROC | PR | ROC | PR |
| Cyt | **0.76** | **0.66** | 0.65 | 0.58 | 0.65 | 0.55 | 0.66 | 0.28 | 0.45 | 0.28 |
| ER | **0.70** | **0.79** | 0.23 | 0.31 | 0.55 | 0.40 | 0.34 | 0.42 | 0.44 | 0.38 |
| EX | **0.65** | **0.63** | 0.00 | 0.00 | 0.00 | 0.00 | 0.00 | 0.00 | 0.00 | 0.00 |
| Mit | 0.00 | 0.00 | 0.00 | 0.00 | 0.00 | 0.00 | 0.00 | 0.00 | 0.00 | 0.00 |
| Nuc | **0.71** | **0.70** | 0.34 | 0.50 | 0.25 | 0.40 | 0.33 | 0.45 | 0.48 | 0.38 |

The names of compartments are abbreviated as Cyt : Cytosol, ER: Endoplasmic Reticulum, EX : Extracellular Region, Mit :Mitochondria, Nuc: Nucleus. NN-RNALoc (with employing NN on k-mer and distance-based profiles features); SVM-RNALoc (with employing support vector machine on k-mer and distance-based profiles features); XGBoost-RNALoc(with employing extreme gradient boosting on k-mer and distance-based profiles features); LightGBM-RNALoc (with employing light gradient-boosting machine on k-mer and distance-based profiles features).

to note that for NN-RNALoc, the probability of each location for each mRNA is computed, then sorted, and the location with the highest probability is reported as the specific mRNA location. To assign more than one location to an mRNA, a threshold can be considered, and all locations with probabilities greater than the chosen threshold can be assigned to the mRNA sequences. However, in order to compare the results of this method with those of other methods, we assign the most probable location. It is worth mentioning that while there is no approach that outperforms the others for predicting all locations, we intend to integrate several methods to predict locations based on a voting measure in our future study.

**Reviewer Point 1.6** — *The area under receiver operating characteristics curve (AU-ROC) and precision-recall curve (AU-PRC) should be included in the performance metrics.*

**Reply**: We thank the reviewer for this comment. We report the area under the Receiver Operator Characteristic (ROC) curve (AUC-ROC) and the area under the Precision-Recall (PR) curve (AUC-PR). Please see Point 1.3.
