# Supplementary material for: NN-RNALoc: Neural network-based model for prediction of mRNA sub-cellular localization using distance-based sub-sequence profiles
Source: PLoS One. 2023 Sep 14;18(9):e0258793. doi: 10.1371/journal.pone.0258793 (PMC10501558; doi:10.1371/journal.pone.0258793)
Supplement: Attachment — Submitted filename: Response to the reviewers_R3.pdf [file pone.0258793.s005.pdf]

Response to the reviewers

10 May 2023

Manuscript title: NN-RNALoc: neural network-based model for prediction of mRNA sub-cellular localization using distance-based sub-sequence profiles

Manuscript number: PONE-D-21-31782

Revision Version: 3

We thank the reviewer for the critical assessment of our work. In the following, we address the concerns point by point.

Reviewer #4: The localization of messenger RNAs (mRNAs) is a frequently observed phenomenon and a crucial aspect of gene expression regulation. It is also a mechanism for targeting proteins to a specific cellular region. Moreover, prior research and studies have shown the significance of intracellular RNA positioning during embryonic and neural dendrite formation. Incorrect RNA localization, which can be caused by a variety of factors, such as mutations in trans-regulatory elements, has been linked to the development of certain neuromuscular diseases and cancer. In this study, we introduced NN-RNALoc, a neural network-based method for predicting the cellular location of mRNA using novel features extracted from mRNA sequence data and protein interaction patterns. In fact, we developed a distance-based subsequence profile for RNA sequence repres. This work is meaningful in this field. This work can be accepted.

Thank you for recognizing the significance of our work on NN-RNALoc, a neural network-based method for predicting mRNA localization, and for recommending its acceptance. Thank you for taking the time to review our work, we appreciate your feedback and insights.

Reviewer #5: 1. Most figures presented in the paper are pixelized. They can be converted to vectorized ones to improve the resolution.

Thank you for your feedback. We have taken your suggestion into consideration and have regenerated the figures presented in the paper in vectorized format with high resolution as suggested. We hope that the improved quality of the figures enhances the readability and overall presentation of our work.

2.The "Evaluation criteria" section should be placed in the Materials and Methods section instead of Results.

Thank you for bringing this to our attention. We appreciate your feedback and have made the necessary revisions by moving the "Evaluation criteria" section from the Results section to the

end of “Materials and Methods” section.
3. The tables in the paper are not using standard three-line tables. Please use three-line tables instead.

Thank you for your comment regarding the format of the tables presented in our paper. We appreciate your feedback and have revised the table format to adhere to the Plose One template and standard three-line table format.
